# Gradient association between pulmonary tuberculosis and diabetes mellitus among households with a tuberculosis case: a contact tracing-based study

**DOI:** 10.1038/s41598-022-05417-2

**Published:** 2022-02-03

**Authors:** Shengqiong Guo, Shiguang Lei, Jinlan Li, Ling Li, Huijuan Chen, Virasakdi Chongsuvivatwong

**Affiliations:** 1 Guizhou Provincial Center for Disease Control and Prevention, Guiyang, 550004 Guizhou China; 2 Department of Epidemiology, Faculty of Medicine, Prince of Songkla University, Hat Yai, 90110 Songkhla Thailand

**Keywords:** Diseases, Health care, Medical research, Risk factors

## Abstract

Pulmonary tuberculosis (PTB) and diabetes mellitus (DM) remain high morbidity and mortality, especially when they are comorbid with each other. Screening for diabetes mellitus in tuberculosis is essential as the incidence and mortality of DM in the population with PTB are higher than in the general people. We aimed to examine the gradient association of tuberculosis on developing DM, the additional yield and the number needed to screen (NNS) to find a new diabetes case. A cross-sectional study was conducted on 801 tuberculosis cases and 972 household contacts in Guizhou, China, from April 2019 to October 2020. After screening for PTB among contacts, all participants were screened for DM and interviewed. Kendall’s tau-b test and proportional odds logistic regression analysis were applied to identify the gradient associations. Among the 1773 subjects, the additional yield of screening was 21.8%. The NNSs of the non-PTB group, the sputum-culture negative and positive groups were 50, 60 and 113, respectively. The gradient incremental establishment of DM and PTB were positively correlated. The general trend on the gradient of DM significantly increased with the gradient increase of PTB. Age 35 years and over, excessive edible oil intake and DM family history were identified as significant predictors of diabetes. Integrated screening for DM targeted to different gradients of PTB combined with associated factors is necessitated to achieve a higher additional yield.

## Introduction

Tuberculosis (TB), especially pulmonary TB (PTB) and diabetes mellitus (DM) remain high morbidity and mortality, particularly when they are comorbid with each other. TB and DM can collectively lead to severe sequelae and complications, even premature deaths, particularly in some areas with limited medical services in low- and middle-income countries (LMICs)^1^. According to the World Health Organization (WHO), around 422 million people worldwide have diabetes, the majority living in LMICs, and 1.5 million deaths are directly attributed to diabetes each year^2^.

The prevalence of DM is consistently found to be higher among PTB patients than among the general population. In accordance with a systematic review of 13 observational studies, DM was associated with an increased risk of PTB (relative risk = 3.11, 95% CI 2.27–4.26)^3^. In an Indian study, among the 313 PTB subjects, the prevalence of newly diagnosed DM and prediabetes was 32% and the overall prevalence of DM was 30%^4^. In Bangladesh, among the 1910 PTB patients who participated in screening for diabetes, 245 (12.8%) were found to have diabetes and 296 (15.5%) to have prediabetes^5^. In a community-based study from China, 6.3% of PTB cases were estimated to be attributed to DM^6^. Globally, the prevalence of diabetes is reported to be rising, estimated at 9.3% (463 million people) in 2019, projected to grow to 10.2% (578 million people) in 2030 and 10.9% (700 million people) in 2045^7^.

The tremendous increase in the prevalence of DM in developing countries reflects inversely on the successful control of PTB, particularly in some places with a high burden of both diseases^7^. DM contributes to the onset of active PTB and deteriorating PTB treatment outcomes, resulting in delays in sputum conversion, increasing the case fatality rate during treatment and increasing relapse rates of PTB after successful completion of treatment. Conversely, PTB can impair glucose tolerance, causing an increase in insulin resistance, decreased insulin production^8^ and transient hyperglycemia^9^. In addition, the side effects of anti-TB drugs, such as optic neuropathy and hepatic impairment can affect the efficacy of hypoglycemic medications, and might impact the clinical management of DM^10^. The efforts to diagnose, detect, and treat DM might have a beneficial effect on PTB control due to the escalating burden of DM, particularly in China, where the burden of PTB remains high^7^.

PTB has different gradients of severity determined by the results of sputum-culture, which is considered as one of the indicators of the severity of PTB. It is an absolute indicator for anti-TB treatment in order to interrupt the transmission and save the patients’ lives^11^.

Delayed diagnosis of DM can lead to an increase in cardiovascular complications and PTB infections. In China, like in many LMICs, DM cases are often undetected in the communities. Community DM screening is useful in not only disclosing the undiagnosed cases, but also identifying prediabetics who can be prevented from getting into full-blown DM after undergoing proper management.

The efficiency of a screening program can be assessed from ‘additional yield’ and ‘number needed to screen’. The additional yield is the percentage of the newly diagnosed cases by screening divided by a total number of known and new cases of interested disease^12,13^. The number needed to screen (NNS) is the number of people who need to be tested to find one positive case^14^.

Both PTB and DM have their gradients of severity as mentioned above. However, few studies had focused on taking an insight into the gradient association between the two essential diseases. With household contact tracing for PTB, we could obtain a high prevalence of PTB among household contacts and document the DM risk gradients between PTB cases and those without PTB.

Therefore, we conducted a large-scale household contact-tracing study to test this relationship. The DM degree was divided into non-DM, prediabetes, and DM groups, and the PTB gradient was divided into non-PTB, sputum-culture negative and positive PTB groups. We hypothesized that the degree of DM increased with the increase of the PTB gradient. Objective-wise, we aimed to (1) examine the association of PTB gradient on developing DM; and (2) assess the additional yield by screening and the number needed to screen for DM to find a new case. Our findings would provide an insight into assessing the strategies for planning DM control measures integrated with TB control programs for policymakers.

## Results

Based on experts’ review, the content validity of the questionnaire met the standard requirement of rigorous research procedure with an alpha coefficient of reliability of 0.85.

### Sampling and general characteristics

**Figure 1:**
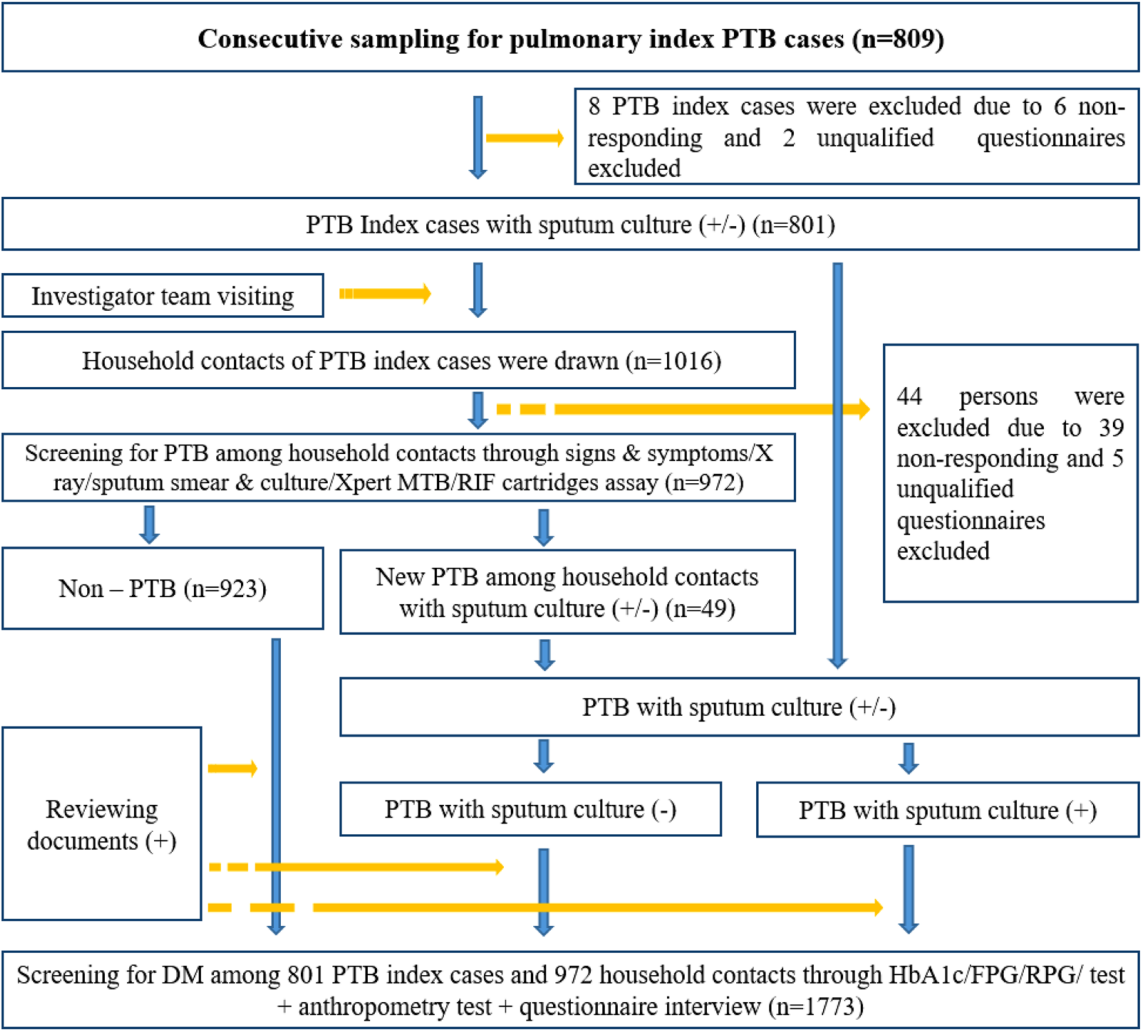
Flow chart of the study in Guizhou, China, 2020. *PTB* tuberculosis, *DM* diabetes mellitus, *HbA1c* glycosylated hemoglobin, *FPG* fasting plasma glucose, *RPG* random plasma glucose.

Overall, 809 index PTB cases were recruited. During the follow-up, six who did not respond and did not participate in the disease screening test were excluded from further analysis. Subsequently, 1,016 adult household contacts were visited, among whom 39 did not respond and therefore did not participate in the disease screening. Two PTB index cases and their five household contacts were also excluded due to unqualified questionnaire information. Eventually, 801 (45.2%) PTB index cases and 972 (54.8%) household contacts were recruited in the study (Fig. 1).

Table 1 compares the background characteristics of the PTB index case group and their household contacts group. The PTB index cases were significantly older, more likely to be male, migrant-laborers, married or cohabitating, and having a lower income. The two groups were not significantly different in terms of religion, ethnicity or level of education attained.

**Table 1:** Socio-demographic characteristics of subjects of the study on the gradient association of tuberculosis on developing diabetes mellitus among households with a tuberculosis case, 2020 (n, %).

Variable	Patient	Contact
**Age group (years)**		
15–34	254 (50.5)	249 (49.5)
35–59	298 (36.6)	516 (63.4)
60–100	249 (54.6)	207 (45.4)
**Gender**		
Female	298 (37.2)	556 (57.2)
Male	503 (62.8)	416 (42.8)
**Religion**		
Buddhism	29 (3.6)	56 (5.8)
Christianity	4 (0.5)	8 (0.8)
Taoism	5 (0.6)	1 (0.1)
Other	44 (5.5)	54 (5.6)
None	719 (89.8)	853 (87.8)
**Ethnicity**		
Han	573 (71.5)	719 (74)
Buyi	107 (13.4)	94 (9.7)
Miao	71 (8.9)	101 (10.4)
Other	50 (6.2)	58 (6.0)
**Education**		
Below primary	368 (45.9)	465 (47.8)
Middle school	351 (43.8)	433 (44.5)
University and above	82 (10.2)	74 (7.6)
**Occupation**		
Clerk	38 (4.7)	41 (4.2)
Student	56 (7.0)	34 (3.5)
Peasant	418 (52.2)	569 (58.5)
Migrant-laborer	289 (36.1)	328 (33.7)
**Marriage**		
Single	174 (21.7)	96 (9.9)
Married/cohabitating	563 (70.3)	843 (86.7)
Separated/divorced/widowed	64 (8.0)	33 (3.4)
**Monthly income (CNY)**		
0–999	365 (45.6)	323 (33.2)
1000–2999	254 (31.7)	406 (41.8)
3000–4999	124 (15.5)	178 (18.3)
5000~	58 (7.2)	65 (6.7)

*CNY* Chinese Yuan.

### Additional yield

Table 2 tabulates the gradient of PTB against that of DM as well as summaries the additional yields and NNSs. In the sixth column of Table 2, the total additional yield was 21.8%, with 25.8% for non-PTB, and 20.0% for both culture (−) and (+) PTB subjects, adding a quarter to one-fifth of the existing known DM cases.

**Table 2:** Prevalence of DM and number needed to screen to find a new DM case among households with a tuberculosis case, 2020 (n, %).

Subject group	Non-diabetes mellitus (a)	Pre-diabetes mellitus (b)	Diabetes mellitus		Additional yield	Number needed to screen	Tau-b value of rank correlation	*P* value of Chi-square test
			Previously known (c)	Newly diagnosed (d)	d/(c + d) × 100 (%)^†^	(a + b + d)/d^#^		
Total	1218	454	79	22	21.8	77	0.066^↑^	< 0.001
Non-PTB	660 (71.5)	232 (25.1)	23 (2.5)	8 (0.9)	25.8	113		
Culture (−) PTB	377 (65.2)	156 (27.0)	36 (6.2)	9 (1.6)	20.0	60		
Culture (+) PTB	181 (66.5)	66(24.3)	20 (7.4)	5 (1.8)	20.0	50		

^†^Additional yield: the percentage of the newly diagnosed cases by screening divided by a total number of known and new cases of interested disease.

^#^Number needed to screen (NNS): the number of people who need to be tested in to find one positive case.

^↑^*P* value < 0.001.

### Number needed to screen (NNS)

The seventh column of Table 2 indicates how many people in each group needed to be screened to find one new DM case. The NNSs of the two PTB groups were approximately half that of the non-PTB one (50 and 60 versus 113).

### Gradient association between PTB and DM

The cross-tabulation of the results for the gradients of PTB (in row) and DM (column a, b, and c + d), as well as the value of Kendall’s tau-b is shown in Table 2. According to the chi-square test, the prevalence of DM among culture (+) PTB cases (9.2%, 25/272) was higher than that among the culture (−) PTB (7.8%, 45/578) and the non-PTB (3.4%, 31/923) subjects. And that of prediabetes was 24.3% (66/272), 27.0% (156/578) and 25.1% (232/923), respectively, among the three groups.

We combined previously known DM and newly diagnosed DM to compute the correlation between the gradients of PTB and DM. Kendall’s tau-b was 0.066 (*P* value < 0.001), indicating a very weak but statistically significant positive rank correlation between the gradients of DM and PTB. In other words, the general trend on the gradient of DM increased with the gradient increase of PTB.

### Univariate analysis results

DM was taken as the dependent variable, and the chi-square test or Fisher's test was used to conduct univariate analysis on the related variables of socio-demographic, behavioral and clinical characteristics. The univariate analysis results suggested that the PTB gradient, older age, family history of DM and hypertension were the significant factors manifested in “Supplementary Material” (Supplementary Table S1).

### Proportional odds logistic regression (POLR) analysis results

Table 3 summarizes the POLR models with covariates incrementally added. The successively added variables were gender, age, ethnicity, occupation, smoking, excessive edible oil intake and DM family history, labeled with the symbol “+” when added in each model, subsequently to the principal hypothesis variable, the gradient of PTB from the Models 2 to 8. The AIC in the last row indicates the level of the fitting. The AICs from all the models except model 9 contain the gradient of PTB severity as it is the principal hypothesis. Model 2 to 5 had demographic characteristics incrementally added. Model 6 and 7 added the subject’s risk behaviors and model 8 added family history of DM.

**Table 3:** Proportional odds logistic regression analysis to choose the best model for assessing the gradient association of tuberculosis on developing diabetes mellitus among households with a tuberculosis case, 2020.

Subject group	Model 1	Model 2	Model 3	Model 4	Model 5	Model 6	Model 7	Model 8	Model 9
Gradients of PTB (principal hypothesis)	+	+	+	+	+	+	+	+	−
Gender	−	+	+	+	+	+	+	+	+
Age	−	−	+	+	+	+	+	+	+
Ethnicity	−	−	−	+	+	+	+	+	+
Occupation	−	−	−	−	+	+	+	+	+
Smoking	−	−	−	−	−	+	+	+	+
Excessive edible oil intake	−	−	−	−	−	−	+	+	+
DM family history	−	−	−	−	−	−	−	+	+
AIC	2728.343	2729.352	2702.068	2697.633	2678.292	2679.988	2667.876	2658.426	2661.744

Excessive edible oil intake: intake of edible oil over 30 g/day/adult. Smoking: smoking in the past 12 months, including both daily and non-daily smoking.

Eventually, to check whether the principal hypothesis still worked, we removed the PTB gradient in model 9 to compare specially with Model 8, which presented the lowest AIC (2658.426) to test which one would fit better when the principal hypothesis was the absence or presence of the PTB gradient. As seen, the AIC of model 8 was lower than that of model 9 (2661.744). Model 8 remained the one with the lowest AIC. Thus, our hypothesis is supported by the data analysis even though we had put some other explanatory variables.

Model 8 was chosen as the best model with the lowest AIC value as 2658.426. The effects of independent variables included in this model are displayed in Table 4 as the adjusted ordinal odds ratios.

**Table 4:** Proportional odds logistic regression analysis for assessing the gradient association of tuberculosis on developing diabetes mellitus among households with a tuberculosis case, 2020 (n, %).

Variable	Result group of screening			Adjusted ordinal OR (95% CI)	*P* value (Wald)	*P* value of LR test with related variable (−)
	Non-DM	Prediabetes	DM			
Total	1218	454	101			
**PTB gradient (principal hypothesis)**						
Non-PTB	660 (71.5)	232 (25.1)	31 (3.4)	Ref	Ref	0.025
Culture (−) PTB	377 (65.2)	156 (27.0)	45 (7.8)	1.33 (1.06, 1.68)	0.015	
Culture (+) PTB	181 (66.5)	66 (24.3)	25 (9.2)	1.35 (1.00, 1.82)	0.050	
**Gender**						
Male	600 (70.3)	214 (25.1)	40 (4.7)	Ref	Ref	0.195
Female	618 (67.2)	240 (26.1)	61 (6.6)	1.20 (0.91, 1.57)	0.194	
**Age group (years)**						
15–34	384 (76.3)	110 (21.9)	9 (1.8)	Ref	Ref	< 0.001
35–59	553 (67.9)	216 (26.5)	45 (5.5)	1.74 (1.32, 2.31)	< 0.001	
≥ 60	281 (61.6)	128 (28.1)	47 (10.3)	2.60 (1.91, 3.55)	< 0.001	
**Ethnicity**						
Han	873 (67.6)	334 (25.9)	85 (6.6)	Ref	Ref	0.023
Buyi	130 (64.7)	63 (31.3)	8 (4.0)	1.37 (0.98, 1.89)	0.062	
Miao	132 (76.7)	38 (22.1)	2 (1.2)	0.71 (0.47, 1.03)	0.079	
Other	83 (76.9)	19 (17.6)	6 (5.6)	0.76 (0.47, 1.21)	0.263	
**Occupation**						
Clerk	53 (67.1)	21 (26.6)	5 (6.3)	Ref	Ref	< 0.001
Student	73 (81.1)	17 (18.9)	0 (0.0)	0.64 (0.30, 1.33)	0.237	
Peasant	700 (70.9)	247 (25.0)	40 (4.1)	0.75 (0.45, 1.26)	0.261	
Migrant-laborer	392 (63.5)	169 (27.4)	56 (9.1)	1.25 (0.76, 2.09)	0.395	
**Smoking**						
No	787 (69.8)	285 (25.3)	55 (4.9)	Ref	Ref	0.495
Yes	431 (66.7)	169 (26.2)	46 (7.1)	0.91 (0.68, 1.20)	0.495	
**Excessive edible oil intake**						
No	1028 (70.8)	336 (23.1)	88 (6.1)	Ref	Ref	< 0.001
Yes	190 (59.2)	118 (36.8)	13 (4.0)	1.63 (1.27, 2.09)	< 0.001	
**DM family history**						
No	1164 (69.5)	433 (25.8)	79 (4.7)	Ref	Ref	< 0.001
Yes	54 (55.7)	21 (21.6)	22 (22.7)	2.16 (1.39, 3.32)	0.001	

LR test: likelihood ratio test. Excessive edible oil intake: intake of edible oil over 30 g/day/adult. Smoking: smoking in the past 12 months, including both daily and non-daily smoking.

Table 4 provides the gradient effect of PTB and other covariates to the outcome on the risk of getting DM based on model 8 from Table 3. The difference between the presence and the absence of a particular variable, with other variables in common, is analyzed as LR test *P* values in the last column. For the first and the main variable, the risk of DM was significantly higher for both the culture (−) group (adjusted ordinal OR = 1.33, 95% CI 1.06, 1.68) and the culture (+) group (adjusted ordinal OR = 1.35, 95% CI 1.00, 1.82) compared to that of the non-PTB group. The *P* value of the LR test was 0.025, indicating that the PTB gradient significantly contributes to the model in predicting the severity of DM. Gender was not significant as the LR test *P* value was 0.195; nor was smoking (LR test *P* value = 0.495). However, the older the age, the higher the gradient of DM. Those who took excessive edible oil had 1.63 times the odds of developing diabetes compared with those who consumed an average amount of oil. In addition, people with a family history of diabetes have a 2.16 times higher risk of developing diabetes than people without a family history of diabetes.

## Discussion

### Summary of findings

PTB and DM are two essential health issues of worldwide concern. A deep exploration of the gradient influence of PTB on developing DM is beneficial for planning the corresponding measures to control these diseases for policymakers according to the different clinical characteristics of patients. Our study found that the incremental establishment of DM and PTB were positively correlated, which was confirmed by Kendall’s tau-b test and POLR analysis. The general trend on the gradient of DM significantly increased with the gradient increase of PTB. The screening could add a quarter to one-fifth of the existing known DM cases, and the NNSs to find a new DM case for the two PTB groups were around half of that in the non-PTB group. In addition, age 35 years and over, having excessive edible oil intake, and presence of DM family history were identified as significant predictors of developing DM.

### Risk of DM and public health implications

The total prevalence of DM among all subjects (5.7%), which may represent a family with a PTB patient, is lower than that (7.6%) from a survey in the general population^15^. The prevalence of DM among the two PTB groups, whether 7.8% or 9.2%, was slightly higher than the 6% reported in Angola in 2019^16^, 6.2% in Paraguay^17^ and 7.5% in India among PTB cases^18^. On the other hand, it is lower than the 11.9% reported by a study in Eastern Nepal^19^. However, these studies might have subjects with different age and gender distributions. A common factor among all these studies was that the PTB patients and families were from a low socioeconomic group.

Although the association between PTB and DM was very weak, the PTB gradient significantly associated with the degrees of DM from the univariate analysis remained significant even when using POLR analysis to adjust for other explanatory factors, such as gender, age, ethnicity, occupation, smoking, excessive edible oil intake and DM family history. This finding is similar to a previous study in Myanmar^6^. Sputum culture-positive is significantly associated with developing DM. It might be linked to the predominant type of *M. tuberculosis* being the Beijing strain in Guizhou^20^. Beijing strain might contain two genes, ESAT-6- and CFP-10-like proteins^21,22^, which could induce the severe responses of the immunologic substances, such as chemokine 10 (CK10), tumor necrosis factor α (TNF-α)^23^, interferon γ (IFN-γ) and interleukin 17 (IL-17)^24^. These cytokines are the effector molecules, regulating the expression of corresponding genes by activating nuclear transcription factors^25^, thereby regulating the apoptosis of pancreatic β-cells and triggering DM^26^.

For the plausible explanation of this gradient association, some studies also reported a relationship between DM and PTB at both the epidemiological and molecular levels. The multidrug-resistant TB, which was the most successful pathogen, could subvert the host immune response and glycemic metabolism through multiple mechanisms, resulting in insidious diseases, such as DM and other diseases^6^. However, if the PTB patients who do not have DM could be identified as PTB and accept anti-TB treatment timely, they may not develop DM after 3 months of anti-PTB treatment^27,28^.

Like PTB, early detection of DM is of value in preventing complications. The additional yield from DM screening reflects its level of worthiness in families with a PTB member. The higher the additional yield is, the more significant the screening would be. The additional yield estimated in our study (21.8%) is much lower than that of two studies conducted, in India (43.0%)^17^ and in Nigeria (59.0%)^10^. Likewise, the NNS in our study was higher than that in those countries (77 in our study, 11 in India and 18 in Nigeria). Our lower additional yield and higher NNS may reflect better access to DM screening in our population than in the countries; it might also be because of some differences in dietary habits and cultural customs^29^. Yet, the screening in this population is worthy of the area where PTB is common. DM screening among households of PTB cases could add a quarter to one-fifth of the existing known DM cases, which indicates an alarming signal that DM should be investigated as routine in PTB screening programs^14^.

The prevalence of prediabetes in this study is noteworthy, which expressed a considerably high prevalence as a quarter in all groups. This means that there will be a large proportion of DM in the future among these family members. According to a study in Germany, prediabetes was observed to demonstrate a predisposition of 29.3% to develop DM after around 10 years, although with 22.5% reverting to normal glucose regulation^30^. Furthermore, even in the prodromal stage, after controlling the development of diabetes, prediabetes is also related to some dysfunction or disability, such as increased chair time, decreased walking speed, and accelerated disability progression^31^. According to a Japanese study, compared with regular blood glucose, prediabetes was associated with a higher risk of all-cause death and death from cancer, but not death from cardiovascular disease. The results were materially unaltered when prediabetes was defined according to three kinds of diagnostic criteria^32^. The high prevalence of prediabetes in our subjects indicates that public health services should be appropriately allocated to the control of prediabetes.

### Other associated factors

Those aged 35 years and above were 1.75–2.59 times more likely to develop DM than those below 35 years, which is consistent with a previous study^33^. Older age was reported as an associated factor for DM, which may be related to decreased immune status in older individuals, making them more susceptible to developing DM^1^. However, this result does not mean that the relevant health services targeted to young people should be ignored. On the contrary, about one-fifth of participants aged between 15 and 35 years suffered from prediabetes, higher than 2.3% diagnosed through the HbA1c test only among teenagers in Canada^34^. The increasing trend was observed in the light of a study during 2002–2012^3^. In that study, the overall unadjusted incidence rates of type 2 diabetes increased by 7.1% annually.

Participants with a family history of DM were 2.15 (95% CI 1.38, 3.13) times more likely to develop DM than those without, which may be related to familial predisposition due to holding similar genes. Growing evidence in some countries based on genetic research has emerged to support this hypothesis^35^. A study from China suggested that two regions, on chromosomes 6q21-q23 and 1q21-q24 respectively, present a significant linkage to type 2 diabetes/impaired glucose homeostasis in Chinese. Another study in China found that INAFM2 rs67839313_T was associated with increased type 2 diabetes risk and FPG levels in Chinese individuals^36^.

The positive relationship between excessive edible oil intake and DM is plausible as absorption of more lipids results in overweight, which has been shown to be a risk factor of DM^37^. In addition, in the present study, compared to Han ethnicity, people from the Buyi ethnic minority group were 1.37 (95% CI 0.98, 1.90) times more likely to develop DM, which might be due to the different lifestyle and cultural customs^38^. In our study, no gender difference was found, which is different from that in the previous studies in India^39^ and Zambia^40^.

Overall, with the potential confounders incrementally included in the various models, the odds ratios of PTB gradient on DM risk were persistently significant. Thus, no potential confounders were evidenced.

### Limitations

There are some limitations in this study. First, we included only index PTB patients and their household contacts. The level of association in this study may not fully reflect that level in the general population. Thus, we could not make a direct comparison of the DM prevalence between index PTB cases and the general population. Second, asymptomatic PTB subjects might be missed from the data due to the way of screening of symptoms and signs first, which is usually performed. Finally, this is a cross-sectional study, which is difficult to identify the exact chronological association of PTB and DM.

## Conclusion

The general trend on the gradient of DM increased with the gradient increase of PTB. Integrated screening for diabetes targeted to different gradients of PTB combined with the associated factors is necessitated to achieve a higher additional yield.

## Methods

### Study design

A PTB contact tracing-based cross-sectional study was conducted in Guizhou Province from April 1, 2019 to October 30, 2020. Eleven out of 88 counties/districts were randomly selected as the study sites.

### Relevant definitions

An index PTB case: a person who has been diagnosed with PTB by at least two positive results of sputum smear, or positive result of one sputum smear with Chest X-rays positive subsequent to 2 weeks of antibiotic medication, or positive result using Xpert MTB/RIF cartridges assay^41,42^, or one sputum sample cultured containing bacilli.

A household contact: a person who was in the same house with a PTB index patient > 6 h per week^43^ from 3 months before the diagnosis and 14 days after the initiation of anti-tuberculosis treatment of the PTB case.

PTB gradients (applied for both the PTB index cases and the contacts): (1) non-PTB = negative Chest X-rays and negative results of sputum exam; (2) culture negative = positive smear and/or Chest X-rays but negative sputum-culture; and (3) culture positive = positive sputum-culture.

DM gradients: (1) non-DM: fasting plasma glucose (FPG) < 110 mg/dl; (2) prediabetes: glycosylated hemoglobin (HbA1c) value between 5.7 and 6.4% (39 ± 46 mmol/mol), or FPG at least 110 mg/dl but below 126 mg/dl^44^ according to the parameters set by the American Diabetes Association (2016); and (3) DM: HbA1c ≥ 6.5% (48 mmol/mol)^45,46^, or FPG ≥ 126 mg/dl, or random plasma glucose (RPG) ≥ 200 mg/dl or a previous diagnosis of DM^44^.

Excessive edible salt intake: according to the Dietary Guidelines for Chinese Residents^47^, more than 6 g/day/adult.

Excessive edible oil intake: according to the Dietary Guidelines for Chinese Residents^47^, more than 30 g/day/adult.

Smoking: smoking cigarettes in the past 12 months, including daily and non-daily smoking.

Alcohol drinking: drinking alcohol in the past 12 months, including daily and non-daily drinking.

### Ethical consideration

The protocol was approved by the Institutional Ethics Committee of the Faculty of Medicine, Prince of Songkla University, Hat Yai, Thailand (No: 61-335-18-1), and the Ethics Committee of Guizhou Provincial Centre for Disease Prevention and Control (No: Q2019-01) before the study was conducted.

### Guidelines and regulations statement

We confirm that all the methods in this article were carried out per relevant human guidelines and regulations.

### Consent to participate

Written informed consent was obtained from each participant before the study was conducted. For those participants aged < 18-year-old, the information sheets were sent to their parents or legal guardians. All the investigations related to them could be started only in the case that their parents or legal guardians had permitted and signed the written informed consent.

### Study procedure and data collection

Initially, newly diagnosed PTB index cases aged ≥ 15 years who have been currently treated for 0–6 months and notified to the national tuberculosis program system from the research locations were retrieved consecutively. Those who were on their retreatment regimen for PTB, pregnant, mentally retarded, or living alone were excluded from this study.

During the eligible patients’ monthly visits to the hospital for medications, TB health care staff would contact the patients to make an appointment with patients for home visits.

During the home visits, we surveyed 1–3 household contacts per household covering all-age in-home visits. Kish’s method was employed to choose the respondents when the number of adult household contacts exceeded three^48^. Although we investigated the all-age household contacts to detect PTB, only those aged ≥ 15 years old were included in the analysis related to DM in this report.

The screening for PTB among the household contacts was conducted following the guideline recommendations of the WHO^49^. First, during home visits, contacts were initially screened for PTB-related symptoms, including cough, expectoration, bloody sputum, or hemoptysis for more than 2 weeks, or fever for more than 2 weeks, and other symptoms as night sweats, fatigue, and chest pain, etc. The participants with the positive symptom results were then referred to the local hospitals to do an Xpert MTB/RIF cartridges assay where available, or Chest X-rays, sputum examinations for acid-fast bacilli and sputum culture. For sputum testing, three expectorated sputum specimens were collected and detected for smear or culture, including spot sputum, night sputum, and morning sputum. PTB was diagnosed if the participant had at least one initial sputum sample containing acid-fast bacilli; or positive chest radiograph not resolved after 2 weeks of broad-spectrum antibiotics treatment^14^.

DM detecting was performed through FPG/RPG or HbA1c. The newly diagnosed subjects with DM and/or PTB were then transferred to the relevant hospitals to receive further treatments by the health system staff.

### Sample size calculation

We used the two independent proportion formula to obtain the minimum sample size using a continuity correction.

$$\begin{aligned} & n_{1} = \left[ {\frac{{z_{{1 - \frac{\alpha }{2}}} \sqrt {\overline{p} \overline{q} \left( {1 + \frac{1}{r}} \right)} + z_{1 - \beta } \sqrt {p_{1} q_{1} + \frac{{p_{2} q_{2} }}{2}} }}{\Delta }} \right]^{2} \\ & r = \frac{{n_{2} }}{{n_{1} }},\;q_{1} = 1 - p_{1} ,\;q_{2} = 1 - p_{2} \\ & \overline{p} = \frac{{p_{1} + p_{2} r}}{1 + r},\;\overline{q} = 1 - \overline{p} \\ \end{aligned}$$

where n_1_ is the number of the PTB cases, including the cases with culture (−) and culture (+) among PTB index cases and among household contacts screened as positive PTB; n_2_ is the number of the non-PTB subjects, including the household contacts screened as negative PTB; the ratio = n_2_/n_1_ = 2.0; p_1_ = 12.5%, the assumed prevalence of DM for the PTB groups, which was considered as 1.56 times higher than that of the non-PTB group; p_2_ = 8.0%, the assumed DM prevalence of the non-PTB group, which is slightly higher than that of an epidemiological survey on DM recorded as 7.6% in Guizhou in 2010^15^; the type I error rate (α) = 0.05; and the type II error rate (β) = 0.20. The formula resulted in n_1 =_ 553, n_2_ = 1,106. With the consideration of 10% of non-response, ultimately, 1843 subjects were planned for recruitment.

### Statistical analysis

Data obtained from the questionnaires and medical record review were entered into EpiData version 3.1 (http://www.epidata.dk/), and R version 3.6.3 (https://cran.r-project.org/) was employed for the statistical analysis. Descriptive statistics was used for general characteristics of the participants, reporting frequencies, proportions, means, medians, and ranges where appropriate. Student's t-test or ANOVA test was employed for age, FPG/RPG and SBP/DBP comparison between/among groups as appropriate, which were summarized using the mean and standard deviation. When the data were not normally distributed, the nonparametric Mann–Whitney U test was performed on the continuous variables. Chi-square tests were used for categorical variables, such as prevalence, percentage, and ratio comparisons. When the expected cell size was less than five observations, Fisher's exact test was performed on discrete variables.

The additional yield of screening DM was calculated as the number of newly diagnosed DM/(previously known + newly diagnosed DM) × 100%^14^. The NNS to find a new DM case was calculated as one divided by the prevalence of newly diagnosed DM after excluding self-reported previously known cases of DM^50^.

Kendall’s tau-b test was employed to assess the gradient association between PTB and DM. Once this relationship was established, we used proportional odds logistic regression (POLR) models to examine predictors of increased risk for DM.

Univariate analysis was used to identify the significant factors associated with DM. Then, with the DM level as the dependent variable, PTB gradient as the principal hypothesis, and the positive factors from the univariate analysis as the covariables, different POLR models were established. Akaike’s Information Criterion (AIC)^51^ was computed to reflect the level of model fitting for adding each variable in each model. The model with the lowest AIC value was considered as the best fitting.

Once we had chosen the best model, the Wald test was conducted to check whether the difference from the variable referent levels was significant. Each independent category was evaluated whether it significantly contributed to the model using the likelihood ratio (LR) test.

## Supplementary Information

Supplementary Table S1.
