# Supplementary material for: Gradient association between pulmonary tuberculosis and diabetes mellitus among households with a tuberculosis case: a contact tracing-based study
Source: Sci Rep. 2022 Feb 3;12:1854. doi: 10.1038/s41598-022-05417-2 (PMC8814182; doi:10.1038/s41598-022-05417-2)
Supplement: Supplementary file 1 — Supplementary Table S1. [file 41598_2022_5417_MOESM1_ESM.pdf]

Title Page

Title:

Gradient association between pulmonary tuberculosis and diabetes mellitus among households with a tuberculosis case: a contact tracing-based study

Authors:

Shengqiong Guo, PhD [1,2], Shiguang Lei, BA[1,**,†], Jinlan Li, MA[1,***], Ling Li, BA[1], Huijuan Chen, MA[1], Virasakdi Chongsuvivatwong, MD, PhD[2,*,†]

[1] Guizhou Provincial Center for Disease Control and Prevention, 550004, Guiyang, Guizhou, China

[2] Department of Epidemiology, Faculty of Medicine, Prince of Songkla University, 90110, Hat Yai, Songkla, Thailand

✉Email: Virasakdi Chongsuvivatwong, cvirasak@medicine.psu.ac.th; Shiguang Lei, 948488961@qq.com; Jinlan Li, 740820442@qq.com.

Supplementary Table S1

Supplementary Table S1. Univariate analysis for the study on gradient association of pulmonary tuberculosis on developing diabetes mellitus, 2020

Variable	Non-DM	Pre-DM	DM	*P* value
**Total**	1218	454	101	
**TB gradient**				< 0.001
Non-PTB	660 (71.5)	232 (25.1)	31 (3.4)	
Culture (-) PTB	377 (65.2)	156 (27.0)	45 (7.8)	
Culture (+) PTB	181 (66.5)	66 (24.3)	25 (9.2)	
**Gender**				0.154
Female	600 (70.3)	214 (25.1)	40 (4.7)	
Male	618 (67.2)	240 (26.1)	61 (6.6)	
**Age (year old)**				< 0.001
15~34	384 (76.3)	110 (21.9)	9 (1.8)	
35~59	553 (67.9)	216 (26.5)	45 (5.5)	
60~100	281 (61.6)	128 (28.1)	47 (10.3)	
**Ethnicity**				0.005
Han	873 (67.6)	334 (25.9)	85 (6.6)	
Buyi	130 (64.7)	63 (31.3)	8 (4.0)	
Miao	132 (76.7)	38 (22.1)	2 (1.2)	
Other	83 (76.9)	19 (17.6)	6 (5.6)	
**Occupation**				< 0.001
Clerk	53 (67.1)	21 (26.6)	5 (6.3)	
Student	73 (81.1)	17 (18.9)	0 (0.0)	
Peasant	700 (70.9)	247 (25.0)	40 (4.1)	
Migrant-labor	392 (63.5)	169 (27.4)	56 (9.1)	
**Education**				0.545
Below primary	561 (67.3)	223 (26.8)	49 (5.9)	
Middle school	543 (69.3)	199 (25.4)	42 (5.4)	
University and above	114 (73.1)	32 (20.5)	10 (6.4)	
**Marital status**				0.001
Single	203 (75.2)	62 (23.0)	5 (1.9)	
Married/cohabitating	959 (68.2)	362 (25.7)	85 (6)	
Separated/divorced/widowed	56 (57.7)	30 (30.9)	11 (11.3)	
**Monthly income (CNY)**				0.770
0~999	476 (69.2)	177 (25.7)	35 (5.1)	
1,000~2,999	450 (68.2)	169 (25.6)	41 (6.2)	
3,000~4,999	201 (66.6)	83 (27.5)	18 (6)	
5000~	91 (74.0)	25 (20.3)	7 (5.7)	
**BMI**				0.060

Norm weight	179 (69.1)	72 (27.8)	8 (3.1)	
Underweight	853 (69.9)	299 (24.5)	69 (5.7)	
Overweight	150 (63.6)	69 (29.2)	17 (7.2)	
Obesity	36 (63.2)	14 (24.6)	7 (12.3)	
**Smoking**				0.115
No	787 (69.8)	285 (25.3)	55 (4.9)	
Yes	431 (66.7)	169 (26.2)	46 (7.1)	
**Drinking alcohol**				0.522
No	827 (69.1)	307 (25.6)	63 (5.3)	
Yes	391 (67.9)	147 (25.5)	38 (6.6)	
**Excessive salt intake**				0.011
No	925 (70.7)	314 (24.0)	70 (5.3)	
Yes	293 (63.1)	140 (30.2)	31 (6.7)	
**Excessive edible oil intake**				< 0.001
No	1028 (70.8)	336 (23.1)	88 (6.1)	
Yes	190 (59.2)	118 (36.8)	13 (4.0)	
**Regular serving vegetable fruit**				0.726
No	243 (70.0)	87 (25.1)	17 (4.9)	
Yes	975 (68.4)	367 (25.7)	84 (5.9)	
**Regular serving meat**				0.692
No	271 (67.1)	110 (27.2)	23 (5.7)	
Yes	947 (69.2)	344 (25.1)	78 (5.7)	
**Regular exercising**				0.235
No	1186 (68.7)	445 (25.8)	96 (5.6)	
Yes	32 (69.6)	9 (19.6)	5 (10.9)	
**Stay-up-late**				0.133
No	1115 (68.2)	428 (26.2)	91 (5.6)	
Yes	103 (74.1)	26 (18.7)	10 (7.2)	
**Family history of DM**				< 0.001
No	1164 (69.5)	433 (25.8)	79 (4.7)	
Yes	54 (55.7)	21 (21.6)	22 (22.7)	
**Family history of HTN**				0.013
No	1044 (69.7)	377 (25.2)	76 (5.1)	
Yes	174 (63.0)	77 (27.9)	25 (9.1)	
